# Optical Coherence Tomography Angiography-Based Quantitative Assessment of Morphologic Changes in Active Myopic Choroidal Neovascularization During Anti-vascular Endothelial Growth Factor Therapy

**DOI:** 10.3389/fmed.2021.657772

**Published:** 2021-05-07

**Authors:** Yao Wang, Zhongli Hu, Tiepei Zhu, Zhitao Su, Xiaoyun Fang, Jijian Lin, Zhiqing Chen, Zhaoan Su, Panpan Ye, Jian Ma, Li Zhang, Jinyu Li, Lei Feng, Chuan-bin Sun, Zhiyong Zhang, Xingchao Shentu

**Affiliations:** ^1^Eye Center of the Second Affiliated Hospital, School of Medicine, Zhejiang University, Hangzhou, China; ^2^Department of Ophthalmology, Zhuji People's Hospital of Zhejiang Province, Zhuji, China

**Keywords:** myopic choroidal neovascularization (mCNV), optical coherence tomography angiography (OCTA), skeletonization process, anti-vascular endothelial growth factor (VEGF) therapy, vessel junctions, quantitative biomarker

## Abstract

**Purpose:** To establish quantitative profile of the morphologic changes among patients with active myopic choroidal neovascularization (mCNV) before and after anti-vascular endothelial growth factor (VEGF) therapy using optical coherence tomography angiography (OCTA) to assess the therapeutic response.

**Methods:** Patients with active mCNV who received anti-VEGF injections between February 2017 to October 2020 and fit the study criteria were retrospectively reviewed. Quantitative analysis of their OCTA images were carried out to evaluate the morphologic features and vascular changes of mCNV lesions in response to anti-VEGF therapy. For further quantitative profiling, mCNV area, fractal dimension, vessel area, vessel density, vessel diameter, vessel length, vessel junction, junction density, and vessel tortuosity were obtained by means of advanced skeletonization postprocessing analyses.

**Results:** Thirty-one eyes of 29 consecutive patients with OCTA-positive mCNV lesions (mean spherical equivalent: −12.55 ± 3.24 diopters) were included. The 31 cases were divided into two phenotypes at baseline: organized interlacing pattern (83.87%) and disorganized vascular loops pattern (16.13%). The values of mCNV area, fractal dimension, vessel area, vessel length, vessel junction, and junction density decreased remarkably 1 month after the initial anti-VEGF injection (*p* < 0.001). Although, vessel density, vessel diameter, and vessel tortuosity increased meanwhile, only vessel diameter displayed statistical significance (*p* = 0.027). Of note, relative ratio analysis showed that vessel junction was the most sensitive biomarker in response to anti-VEGF therapy, reflecting a mean decrease of 50.36%. Sensitivity lowered successively in biomarkers of vessel length, vessel area, junction density, mCNV area, and fractal dimension. In addition, percent change of mCNV area (*r* = 0.552, *p* = 0.002), fractal dimension (*r* = 0.446, *p* = 0.017), vessel area (*r* = 0.518, *p* = 0.005), and vessel length (*r* = 0.440, *p* = 0.019) were moderately associated with that of central retinal thickness.

**Conclusions:** The study showed morphological as well as quantitative changes on OCTA responding to anti-VEGF treatment in mCNV patients, among which vessel junctions might be the most predictive biomarker. OCTA-based analysis, providing intuitive images and a large spectrum of quantitative data at the same time, could promote new insights into the therapeutic response assessment in mCNV patients.

## Introduction

It was estimated by World Health Organization that, by 2030, myopia would affect 3.36 billon people globally and present as the leading cause of vision impairment (1, 2). The number of people with high myopia projected from 399 million in 2020 (5.2% of global population) to 517 million in 2030 (6.1% of global population) (3). High myopia is defined as spherical equivalent (SE) more than −6 to −8 diopters in the context of eye elongation (ocular axial length ≥26.0–26.5 mm). Elongation of the axial length and posterior staphyloma drives the development of pathologic myopia (4). Pathologic myopia brings further irreversible vision challenges with possible complications including glaucoma, retinal detachment and myopic macular degeneration.

Myopic choroidal neovascularization (mCNV) originates in the choroid and distorts retinal anatomy and is normally classified as type 2 CNV, which is a common vision-threatening complication secondary to pathologic myopia. Approximately 5–11% of individuals with pathologic myopia will develop mCNV, especially those with patchy retinal atrophy, lacquer cracks and choroidal thinning. Development of chorioretinal atrophy around the regressed untreated-CNV lesion will lead to atrophic myopic maculopathy and poor vision prognosis (4–6). In recent years, the favorable safety and therapeutic outcomes promoted anti-vascular endothelial growth factor (VEGF) to be first-line treatment for subfoveal and juxtafoveal mCNV. Common practice includes a single intravitreal anti-VEGF injection (IVI), with a pro re nata (PRN) regimen during follow-up (4, 5).

Optical coherence tomography (OCT) has been successfully and widely used to diagnose and monitor treatment response in mCNV. Some OCT-based structural features have been recognized as traditional indicators of mCNV activity, such as retinal thickness, intraretinal fluid, and subretinal fluid (7, 8). Such biomarkers mainly reflect the secondary fluid-related consequences of CNV activity but lack direct evaluation of vascular pathology, let alone visualization of the choriocapillaries (9). Fluorescein angiography (FA), the gold standard to identify the presence and activity of mCNV, can also compromise details of neovascular network due to dye leakage (4, 10). Recently, the introduction of OCT angiography (OCTA) revolutionarily enables direct and meticulous visualization of CNV morphology through a non-invasive and dye-less approach, and enables quantitative assessment of neovascular structure and treatment response based on high-quality layered images (4). The sensitivity of OCTA to identify mCNV is 90–94% and the specificity is 93.75% (11–13). Previous literatures had reported qualitative manifestations of mCNV regression after anti-VEGF therapy in OCTA (14, 15), but only a few studies have described the quantitative changes. Among the quantitative investigators, Cheng et al. (15, 16) reported that significant decreases in mCNV area and flow area after one-month therapy of mCNV with ranibizumab or conbercept. However, OCTA parameters other than mCNV area or flow area were rarely discussed to reflect mCNV activity. As far as we are concerned, the morphological and quantitative data of OCTA had been successfully applied in patients with neovascular age-related macular degeneration (nAMD) to evaluate CNV activity, and treatment response, with thorough analysis on junction number/density, endpoint number/density, vessel length, etc., (17, 18). Therefore, it is reasonable to predict great scientific significance in an intensive quantitative investigation of mCNV with OCTA.

This study was designed to profile the quantitative changes of mCNV lesion during its most sensitive phase, namely the one dose loading phase, using OCTA and postprocessing technique, and to explore biomarkers from OCTA that could sensitively reflect the outcome of VEGF inhibition on the neovascular biology.

## Materials and Methods

This was a retrospective case series review of 31 eyes of 29 consecutive patients with mCNV who visited the Eye Center of the Second Affiliated Hospital, School of Medicine, Zhejiang University between February 2017 and October 2020. They received at least one dose injection of anti-VEGF agent (Conbercept 0.5 mg/0.05 ml or Ranibizumab 0.5 mg/0.05 ml) with at least a 6-month follow-up. This study followed the tenets of the Declaration of Helsinki and was approved by the Ethics Committee of the Second Affiliated Hospital, School of Medicine, Zhejiang University. Because of the retrospective nature of the study, patient consent for inclusion was waived.

### Inclusion Criteria

The inclusion criteria were as follows: (1) myopia with spherical equivalent more than −6.0 diopters and/or the ocular axial length >26.5 mm (19, 20); (2) presence of myopic fundus changes as defined by the International Photographic Classification and Grading System for Myopic Maculopathy (2); (3) active and treatment-naïve CNV at baseline; (4) thorough ophthalmic examinations at baseline, including slit-lamp examination, dilated fundus examination with ophthalmoscope, intraocular pressure, refractive status, axial length, fundus photography, spectral-domain OCT (SD-OCT), OCTA, and FA. Activity status of mCNV at baseline was confirmed by FA and OCT. Active mCNV appeared as a dome-shaped elevation with a hyperreflective component above the retinal pigment epithelium with exudative signs including retinal thickening, intraretinal fluid and subretinal fluid on OCT imaging. FA typically revealed hyperfluorescent area in early frames indicative of filling of the neovascular complex, with late leakage into the mCNV area (4, 11). One month after anti-VEGF therapy, every patient was re-examined by OCT and OCTA. All images were reviewed for final inclusion by two independent retina specialists (YW and ZTS). Any discrepancies in the data were resolved through reassessment and discussion with a senior researcher (XYF).

### Exclusion Criteria

We excluded patients with AMD, adult onset foveomacular vitelliform dystrophy, multifocal choroiditis, punctate inner choroidopathy, retinoschisis, or CNV caused by any other causes other than myopia. OCTA images with poor quality, such as projection artifacts from vessels located above the plane of the image or an overly dark image filled with extremely thick outer choroidal vessels, were excluded. Patients with history of intraocular surgery were excluded, such as pars plana vitrectomy.

### Acquisition of Optical Coherence Tomography Angiography

OCTA images were obtained by a commercial SD-OCT system (RTVue-XR; Optovue, Inc., Freemont, CA). The instrument could delineate the microvascular structures of retina and choriocapillaries *via* a split spectrum amplitude-decorrelation angiography. The OCTA device with a light source centered on 840 nm and a bandwidth of 50 nm could operate with two consecutive 304 raster B-scans (each B-scan containing 304 A-scans). An A-scan rate of 70,000 scans per second with motion correction minimized artifacts arising from microsaccades and fixation changes. The OCTA software provided four *en face* images, including superficial capillary plexus, deep capillary plexus, outer retina, and choriocapillaris layers. Initially, the presence of mCNV with clear boundary was assessed on *en face* images generated by the automatically segmented choriocapillaris slab and the outer retina slab. Subsequently, the CUSTOM function in the AngioVue software (version 2018.1.0.43; Optovue, Inc.) was used for manual segmentation to acquire the entire thickness mCNV with the clearest boundary and the least perilesional artifact. The manual adjusted OCTA images were used for subsequent qualitative and quantitative analyses. Manual adjustment of segmentation changed the thickness and axial position of the OCTA slab to minimize inaccuracies in CNV contour (21).

### Qualitative Evaluation of Optical Coherence Tomography Angiography Images

Two investigators independently analyzed the baseline OCTA images. On the basis of the overall appearance, mCNV lesions were classified into two phenotypes: organized interlacing pattern or disorganized vascular loops pattern, as previously reported (4, 11, 16). The morphology of the mCNV was qualitatively described based on five criteria (11, 14): (1) overall pattern, including organized interlacing and disorganized vascular loops patterns. (2) exuberant capillaries, numerous tiny capillary ramifications were typical of a recent lesion. (3) anastomoses and loops. (4) perilesional hypointense halo, local regions of choriocapillaris alteration encircling the CNV lesion. (5) feeder vessel. The well-defined organized interlacing lesions would be further classified into medusa, sea-fan or tree-in-bud patterns: medusa pattern corresponded to the lesion where vessels radiated in all directions from the center; sea-fan pattern corresponded to the lesion where vessels radiated in all directions from one side; tree-in-bud pattern corresponded to the round lesion without obvious vascular trunk (14, 16).

### Quantitative Evaluation of Optical Coherence Tomography Angiography Images

**Figure 1:**
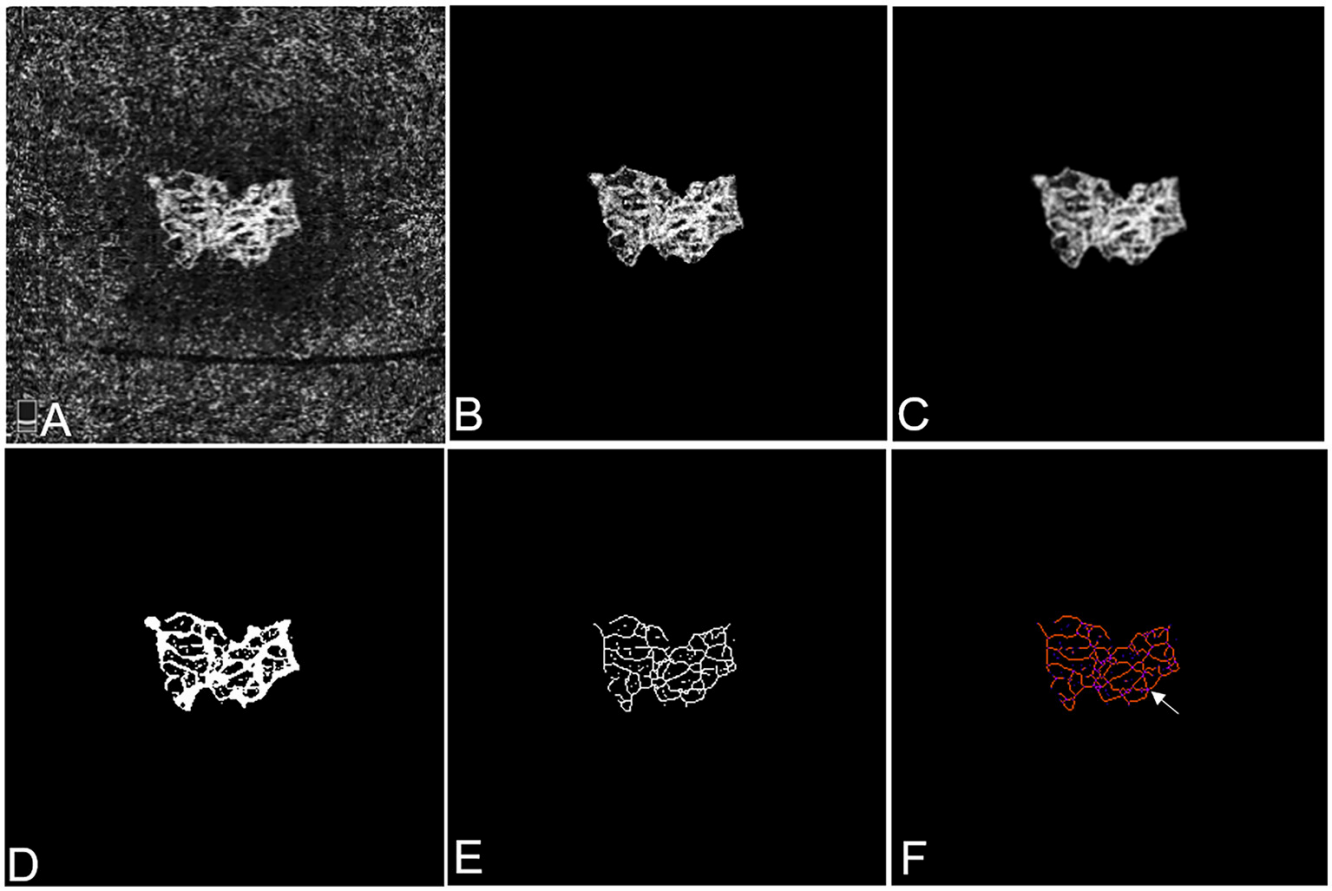
Representative OCTA images of mCNV before and after cropping, binarisation, and skeletonisation in Patient #18, a 60-year-old female (refractive error −15.50 diopters) in the right eye. **(A)** OCTA image showing the entire thickness mCNV lesion after manual adjustment of segmentation. **(B)** The full extent of mCNV was manually delineated, and the mCNV area was measured by counting the pixels contained within the contour. **(C)** The Gaussian kernel was used to reduce the image noise and obtain a smooth image. **(D,E)** The binary OCTA image was formed by Frangi vesselness filter and local adaptive thresholding and used for calculating vessel area, fractal dimension and vessel lengths. Vessel density was calculated using vessel area and mCNV area. **(F)** Tagged Skeleton image was used to calculate the numbers of vessel junctions (white arrow). Junction density was calculated by dividing the vessel junction by the vessel length. Images **(B–E)** were acquired by MATLAB program and image **(F)** was acquired by IMAGE J software.

To establish OCTA biomarkers for mCNV, we developed a CUSTOM MATLAB program (R2020b; MathWorks Inc., Natick, MA) to process OCTA images (Figure 1), as reported in previous studies (22). All OCTA images were binarized before quantitative analysis. Briefly, the boundary of mCNV area was manually outlined for further binarization. Firstly, the mCNV image was denoised using a Gaussian kernel. Then, a combined strategy with Frangi vesselness filter and local adaptive thresholding was applied to produce the final binary OCTA image. The following quantitative mCNV morphologic biomarkers were automatically calculated from both binary image and corresponding skeletonized image: mCNV area, vessel area, vessel length, vessel density, vessel diameter, and fractal dimension.

In addition, vessel junction, junction density, and vessel tortuosity were calculated using the image software (Image J, National Institutes of Health, USA), as described in published literature (23). Central retinal thickness (CRT) was automatically provided by OCT analysis, considered as a traditional OCT-based indicator of treatment response.

The OCTA metrics calculation based on skeletonized binary OCTA images were as follows:

1. mCNV area (mm^2^): A lesion size biomarker that was calculated according to the manually outlined boundary of mCNV, indicating the entire size of mCNV lesion.
2. Vessel area (mm^2^): A vessel size biomarker indicating the size of vessel components with flow signals in the lesion.
3. Vessel density: A vascular biomarker that was calculated as the percentage of the area occupied by vessels in the mCNV lesion.
4. Vessel length (mm): A vascular biomarker calculating the sum of Euclidean distances between the pixels of all the vessels in the CNV lesion, indicating the total neovascular length.
5. Vessel diameter (μm): A vascular biomarker that was calculated as the non-skeletonized vessel area divided by the skeletonized total vessel length, indicating the average vessel caliber of the mCNV.
6. Fractal dimension: A measure representing vessel branching complexity, which was obtained from the skeletonized binary image using the box-counting method. Higher fractal dimension values indicated more complex vessel branching pattern.
7. Vessel junction: A vascular biomarker that was defined as the points of vascular connections, indicating internal branching and anastomotic connections in vascular networks. It reflected the activity of CNV lesion.
8. Junction density (n/mm): A biomarker related to vessel branching complexity was calculated as the vessel junction number per unit vessel length. It could be interpreted as a measure of anastomotic activity in proportion to the total neovascular length, which also reflected the activity of CNV lesion.
9. Vessel tortuosity: A morphologic biomarker that quantified the microtortuosity of the CNV was calculated as the actual length of each branch divided by the imaginary straight length between two branch nodes (24). Smaller tortuosity values indicated straighter CNV vessels.

For subgroup analysis, subjects were further divided into two groups according to number of IVIs during the 6 months of follow-up, including “stable group” (one or two injections) vs. “unstable group” (more than two injections).

### Statistical Analysis

Statistical analyses were performed using SPSS software (Windows version 21, SPSS Inc., Chicago, IL, USA). Descriptive statistics was expressed as mean ± standard deviation (SD). Normality tests of data distribution were assessed by Shapiro-Wilks test. Wilcoxon rank sum test was used to analysis the difference and relative ratio was used to evaluate the degree of variation between the baseline group and post-IVI group. Mann-Whitney *U*-test was used to evaluated the difference between the biomarkers in the “stable group” and “unstable group” at baseline. We used Spearman rank correlation coefficient (*r*, ranged from −1 to +1) (25) to evaluate the correlation between the percent change of OCTA-based biomarkers and CRT. Absolute magnitude of *r* > 0.90 was considered as “very strong correlation,” 0.70–0.89 as “strong correlation,” 0.40–0.69 as “moderate correlation,” 0.10–0.39 as “weak correlation,” and 0.00–0.09 as “negligible correlation.” The significance level for all testing was set at *p* < 0.05.

## Result

### Baseline Clinical Characteristics of the Study Population

Thirty-one eyes (14 right eyes and 17 left eyes) of 29 patients (12 males and 17 females) with mCNV were included in this study. The mean age was 44.48 ± 12.51 years (ranged from 25 to 67 years old), the mean spherical equivalent was −12.55 ± 3.24 diopters (ranged from −6.50 diopters to −18.0 diopters), the mean IVI numbers were 2.19 ± 0.87 (ranged from 1 to 4). 19 and 10 eyes were classified into the stable and unstable groups, respectively. Baseline demographical and clinical characteristics are shown in Table 1.

**Table 1:** Demographic and morphologic characteristics of study population.

**Patient characteristics (*n* = 29)**	
Age, years, mean ± SD (range)	44.48 ± 12.51 (25~67)
Sex (male/female)	12/17
Eye characteristics (*n* = 31)	
Mean SE, diopters, mean ± SD (range)	−12.55 ± 3.24 (−18.00~-6.50)
Right eye	14
Left eye	17
Number of injections, mean ± SD	2.19 ± 0.87

*SE, Spherical Equivalent*.

### Optical Coherence Tomography Angiography Characterization of Myopic Choroidal Neovascularization Morphology at Baseline

**Figure 2:**
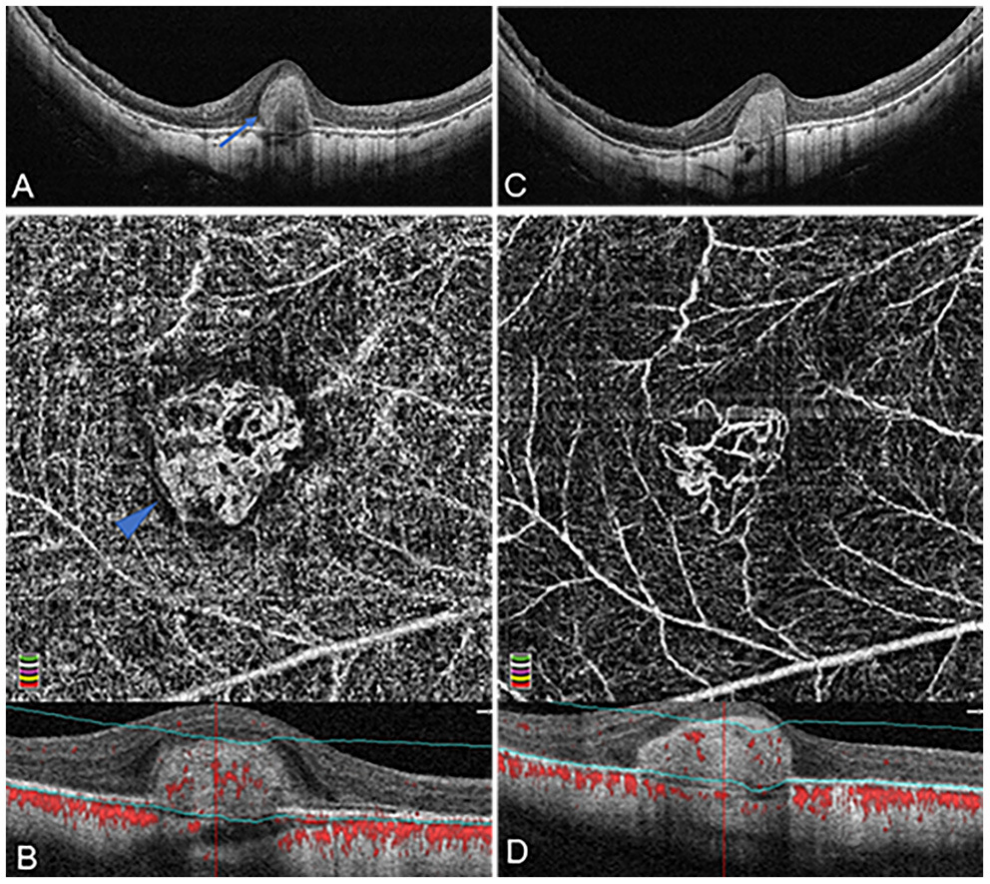
Organized interlacing pattern of active mCNV imaged by OCT and OCTA before and after anti-VEGF injection in Patient #15, a 49-year-old man (refractive error −14.75 diopters) in the right eye. OCTA segmentation was manually adjusted to acquire a clear *en face* image visualizing the entire thickness mCNV lesion. **(A)** Spectral-domain OCT B-scan at baseline showed a typical subretinal hyper-reflective type-2 CNV (blue arrow) with discontinuous retinal pigment epithelium (RPE). **(B)** OCTA *en face* image (3 × 3 mm) at baseline depicted a larger, well-circumscribed, interlacing type of neovascular membrane. This mCNV lesion contained numerous tiny capillary ramifications, anastomoses and loops, which was bordered by a dark halo, showing a medusa shape (blue arrowhead). **(C)** One month after the first injection, OCT image revealed significant shrinkage of CNV lesion with a clearer contour. **(D)** One month after the first injection, OCTA *en face* image (3 × 3 mm) indicated a reduction of CNV size, anastomoses, and perilesional halo, a dramatic attenuation of capillaries and small caliber vessels, and a reservation of large caliber vessels. The lower parts of image **(B,D)** represented the cross-sectional structural OCT images corresponding to the upper OCTA *en face* images, respectively, displaying the boundaries (green lines) of the OCTA slabs after manual adjustment of segmentation.

At the baseline OCTA examination in Table 2, the overall pattern of high-flow neovascular network could be categorized into two phenotypes: organized interlacing pattern and disorganized vascular loops pattern. In the first phenotype (26/31, 83.87%) (Figures 2A,B), OCTA images revealed a larger, well-circumscribed, interlacing type of neovascular membrane. This pattern comprised relatively exuberant capillary ramifications, anastomoses and loops, with a mean selected mCNV area of 0.44 ± 0.54 mm^2^, a mean vessel junction number of 52.92 ± 47.84, and a mean junction density of 7.49 ± 1.69 /mm. Of note, the cases in this subgroup were further classified into medusa (12/26, 46.15%), sea-fan (3/26, 11.54%), and tree-in-bud (11/26, 42.31%) pattern.

**Table 2:** Demographic characteristics and optical coherence tomography angiography features of patients with myopic choroidal neovascularization.

**Pt No**.	**Age, years**	**Sex**	**Eye**	**SE, diopters**	**OP**	**EC**	**AL**	**HH**	**FV**
1	58	F	L	−13.00	OI	Y	Y	N	Y
2	47	M	R	−15.50	OI	Y	Y	Y	N
3	67	F	R	−7.13	OI	Y	Y	Y	Y
4	52	F	R	−13.50	OI	Y	Y	Y	N
5	50	F	L	−12.63	OI	Y	Y	N	N
6	50	M	R	−10.88	OI	Y	Y	Y	Y
7	30	F	L	−13.50	OI	Y	Y	Y	Y
8^+^	51	M	R	NA^++^	OI	Y	Y	N	N
8^+^			L	NA^++^	DVL	N	Y	N	N
9	49	F	L	−13.25	OI	Y	Y	Y	N
10	31	F	L	−6.50	OI	Y	Y	Y	N
11^+^	43	M	R	−7.75	OI	Y	Y	Y	Y
11^+^			L	−9.13	OI	Y	Y	Y	N
12	25	F	L	−8.50	OI	Y	Y	Y	N
13	53	F	L	−8.50	OI	Y	Y	Y	N
14	57	F	R	−17.00	OI	Y	Y	Y	N
15	49	M	R	−14.75	OI	Y	Y	Y	N
16	37	M	L	−8.00	OI	Y	Y	Y	Y
17	35	M	L	−13.50	DVL	N	Y	Y	N
18	60	F	R	−15.50	OI	Y	Y	Y	N
19	30	F	L	−11.75	OI	Y	Y	Y	N
20	62	M	R	−11.25	OI	Y	Y	Y	N
21	28	F	L	−11.13	OI	Y	Y	Y	N
22	31	M	L	−7.00	OI	Y	Y	Y	N
23	34	F	L	−7.63	DVL	N	Y	N	N
24	58	F	L	−12.25	OI	Y	Y	Y	N
25	29	M	R	−9.88	DVL	N	N	N	N
26	35	M	R	−13.75	DVL	N	Y	Y	N
27	46	M	R	−18.00	OI	Y	Y	Y	N
28	27	F	R	−17.00	OI	Y	Y	Y	N
29	66	F	L	−10.75	OI	Y	Y	Y	N

*Pt No., patient number; SE, Spherical Equivalent; OP, Overall Pattern; OI, Organized Interlacing; DVL, Disorganized Vascular Loops; EC, Exuberant Capillaries; AL, Anastomoses and Loops; HH, Hypointense Halo; FV, Feeder Vessel; F, Female; M, Male; R, Right eye; L, Left eye; N, No; Y, Yes; 8^+^ and 11^+^ mean that two eyes of each patient were included; NA^++^ means that we didn't obtain the diopter of eye, but axial length of right eyeball was 30.59 mm and axial length of right eyeball was 29.84 mm*.

**Figure 3:**
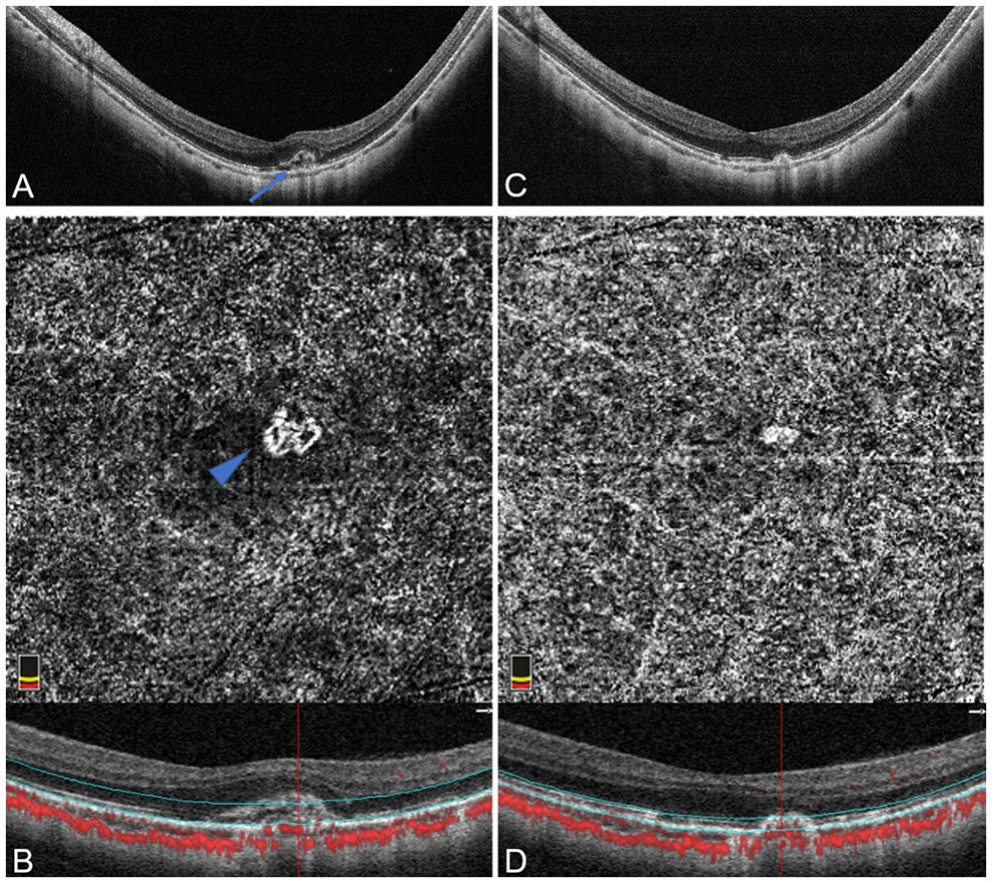
Disorganized vascular loops pattern of active mCNV imaged by OCT and OCTA before and after anti-VEGF injection in Patient # 8, a 51-year-old man (axial length 29.84 mm) in the left eye. OCTA segmentation was manually adjusted to acquire a clear *en face* image visualizing the entire thickness mCNV lesion. **(A)** Spectral-domain OCT B-scan at baseline showed a small hyper-reflective type-2 CNV (blue arrow) beneath the neurosensory retina with fuzzy ellipsoid zone and discontinuous RPE. **(B)** OCTA *en face* image (3 × 3 mm) at baseline detected a small disorganized vascular loop-like lesion with unobvious capillary ramifications, suggesting an immature network (blue arrowhead). **(C)** One month after the first injection, OCT image revealed narrowing of mCNV lesion with a relatively clear ellipsoid zone. **(D)** One month after the first injection, OCTA *en face* image (3 × 3 mm) indicated an absence of vascular details due to the obviously attenuated mCNV lesion. The lower parts of image **(B,D)** represented the cross-sectional structural OCT images corresponding to the upper OCTA *en face* images, respectively, displaying the boundaries (green lines) of the OCTA slabs after manual adjustment of segmentation.

In the second phenotype (5/31, 16.13%) (Figures 3A,B), OCTA images revealed a disorganized vascular loops pattern, consisting of a smaller vascular loop-like lesion, and unobvious capillary ramifications, with a mean selected mCNV area of 0.12 ± 0.09 mm^2^, a mean vessel junction number of 16.67 ± 4.16 and a mean junction density of 7.11 ± 1.73 /mm. The mean values of the aforementioned three biomarkers were higher of the organized interlacing subgroup than that of the disorganized vascular loops subgroup, but the differences were not statistically significant (all *p* > 0.05).

Exuberant capillaries, anastomoses and loops, perilesional hypointense halo, and feeder vessel were identified in 26/31 (83.87%), 30/31 (96.77%), 25/31 (80.65%), and 6/31 (19.35%) cases, respectively. The 26 eyes with exuberant capillaries, and 6 eyes with visualization of feeder vessels were all included in the interlacing pattern subgroup. There were no significant differences between the two subgroups in age, sex, mean spherical equivalent, and injection number (all *p* > 0.05).

### Optical Coherence Tomography Angiography Quantitative Analysis of Myopic Choroidal Neovascularization Lesion Before and After Anti-VEGF Therapy

Twenty-nine eyes were included for quantitative comparisons of the OCTA-based biomarkers associated with mCNV morphology before and after anti-VEGF therapy, as shown in Table 3. Two eyes with unidentifiable mCNV lesion after anti-VEGF therapy were excluded: one patient due to full disappearance of mCNV and in another due to ill-defined boundaries of the mCNV induced by projection artifacts. One month after anti-VEGF therapy, the mCNV size intuitively decreased, and the vessels of mCNV shrunk in OCTA images (Figures 2C,D, 3C,D). Furthermore, the small-diameter vessels decreased or even disappeared, and the main or larger-diameter vessels were still present. The mean values of mCNV area, fractal dimension, vessel area, vessel length, vessel junction, junction density, and CRT decreased, while the mean values of vessel density, vessel diameter and vessel tortuosity increased. We found significant differences in mCNV area, fractal dimension, vessel diameter, vessel area, vessel length, vessel junction, junction density, and CRT between baseline and post-IVI group (all *p* < 0.05). Moreover, the mean values of OCTA-based biomarkers in “stable group” were lower than that in “unstable group” except vessel density and junction density, although, there were no statistical differences.

**Table 3:** Quantitative biomarkers of the optical coherence tomography angiography for myopic choroidal neovascularization.

	**Baseline**	**Post-IVI**	***p-*Value**	**RR (%)**
Quantitative biomarkers, mean (SD)				
mCNV area, mean (SD), mm^2^	0.40 (0.52)	0.28 (0.41)	<0.001	70.00
VA, mean (SD), mm^2^	0.20 (0.20)	0.13 (0.14)	<0.001	65.00
VLD, mean (SD)	0.55 (0.09)	0.56 (0.14)	0.829	102.82
FD, mean (SD)	1.08 (0.15)	0.95 (0.23)	<0.001	87.96
VD, mean (SD), μm	31.11 (3.78)	37.47 (13.94)	0.027	120.44
VL, mean (SD), mm	6.96 (7.92)	4.38 (5.81)	<0.001	62.93
VT, mean (SD)	1.26 (0.07)	1.36 (0.33)	0.276	107.93
VJ, mean (SD)	49.36 (47.43)	24.50 (28.25)	<0.001	49.64
JD, mean (SD), n/mm	7.52 (1.65)	5.09 (2.26)	<0.001	67.69
CRT, mean (SD), mm	316.75(72.72)	257.39(30.66)	<0.001	81.26

*mCNV, Myopic Choroidal Neovascularization; VA, Vessel Area; VLD, Vessel Density; VD, Vessel Diameter; VL, Vessel Length; VT, Vessel Tortuosity; VJ, Vessel Junction; JD, Junction Density; FD, Fractal Dimension; RR, relative ratio; IVI, Intravitreal anti-VEGF Injection*.

Relative ratio was calculated to indicate the percentage of biomarker change after anti-VEGF therapy (Table 3). The mCNV area, fractal dimension, vessel area, vessel length, vessel junction, junction density, and CRT decreased by 30.00, 12.04, 35.00, 37.07, 50.36, 32.31, and 18.74%, respectively, while vessel density, vessel diameter, and vessel tortuosity increased by 1.82, 20.44, and 7.93%, respectively.

### Correlation of Optical Coherence Tomography Angiography-Based Biomarkers and Optical Coherence Tomography-Based Central Retinal Thickness

When the morphological OCTA-based biomarkers and structural therapeutic response expressed by OCT-based CRT were correlated, we found that there was statistically significant moderate correlation between the percent changes of four OCTA-based biomarkers (mCNV area, fractal dimension, vessel area and vessel length) and that of CRT (*r* = 0.552, *p* = 0.002 for mCNV area; *r* = 0.446, *p* = 0.017 for fractal dimension; *r* = 0.518, *p* = 0.005 for vessel area; *r* = 0.440, *p* = 0.019 for vessel length) (Table 4).

**Table 4:** The correlation analysis between the percent change of central retinal thickness and quantitative OCTA-based biomarkers in myopic choroidal neovascularization.

	**PC (CRT)**	
	***r***	***p***
PC (mCNV area)	0.552	0.002
PC (VA)	0.518	0.005
PC (VLD)	−0.251	0.197
PC (FD)	0.446	0.017
PC (VD)	−0.190	0.333
PC (VL)	0.440	0.019
PC (VT)	−0.152	0.467
PC (VJ)	0.357	0.062
PC (JD)	−0.090	0.649

*mCNV, Myopic Choroidal Neovascularization; VA, Vessel Area; VLD, Vessel Density; VD, Vessel Diameter; VL, Vessel Length; VT, Vessel Tortuosity; VJ, Vessel Junction; JD, Junction Density; FD, Fractal Dimension; CRT, Central Retinal Thickness; PC means percent change defined as (post-IVI – baseline)/baseline*.

## Discussion

High myopia is the second leading cause of CNV following nAMD and mCNV brings further irreversible visual impairment. Without treatment, more than 90% of mCNV-affected eyes are likely to experience a progressive blindness within 10 years (26, 27). Traditional structural OCT-based hallmarkers associated with fluid are widely used in nAMD patients to evaluate the treatment response and disease activity in clinical routine, but unlike the CNV in nAMD, mCNV is generally less extensive and exudative. The subjective nature also makes it difficult to reach consensus between readers. With the emerging technique of OCTA, new quantitative biomarkers for a detailed characterization of CNV morphology and objective assessment of treatment response may become available (10, 15, 28). By setting and analyzing the diverse OCTA-based biomarkers, this retrospective study aimed to quantitatively elucidate the mCNV biology and evaluate the therapeutic effect of IVI.

A clear and recognized classification of mCNV phenotypes on OCTA imaging has not been established. The morphology varies considerably associated with diverse factors such as stage and state of CNV lesions, as well as shape of the staphyloma. Querques et al. (28) enrolled 28 eyes with active and inactive mCNV, and defined two phenotypes of interlacing and tangled network on OCTA, which could partly reflect the activity of CNV lesion. Bruyère et al. (11) divided the active mCNV in 20 eyes (treatment-naïve and recurrent) into small disorganized vascular loops and larger organized interlacing pattern correlating with the vascular maturity, age, and treatment status. The mCNV cases included in our study were all active and treatment-naïve at baseline. We have distinguished two subtypes of mCNV similar to those reported by Bruyère et al.. At baseline, the mean mCNV area was 0.44 ± 0.54 mm^2^ and the vessel area was 0.21 ± 0.20 mm^2^, which were quite close to the results of Bruyère et al. The prevailing organized interlacing pattern was found to be larger and more branched than the disorganized vascular loops pattern but the differences were not statistically significant (all *p* > 0.05). Bruyère et al. hypothesized that the combination of morphology and size of the neovascular lesion, was correlated with maturity: while small, disorganized vascular loops pattern suggested an immature status, larger, highly structured interlacing pattern suggested a mature status. The above statuses might represent two sequential stages in the life cycle of mCNV development. Furthermore, mCNV completely vanished on OCTA after one anti-VEGF injection in one case of small disorganized vascular loop-like lesion in our study, which led to the speculation that the immature neovascular network would be more likely to achieve full regression.

In order to further quantitatively evaluate mCNV morphology, MATLAB, and IMAGE J were offered semiautomated analysis of several morphologic and spatial vessel biomarkers to assess the treatment response and the mCNV activity. The OCTA-based biomarkers included the mCNV area, fractal dimension, vessel area, vessel density, vessel diameter, vessel length, vessel tortuosity, vessel junction, and junction density, providing quantitative information about vessel area/density, branching pattern, and uniformity of the CNV microvasculature. The mean values of mCNV area, fractal dimension, vessel area, vessel length, vessel junction, and junction density were statistically different (all *p* < 0.001) between the baseline and post-IVI group. Compared with the baseline group, the six biomarkers decreased with a maximum change of 50.36% for vessel junction and a minimum change of 12.04% for fractal dimension, indicating significant reduction in lesion size, vascular complexity, and CNV activity after anti-VEGF therapy. Myopic CNV area and vessel area were the most frequently mentioned quantitative biomarkers in OCTA studies. Cheng et al. (15) reported that OCTA analyses revealed reduction of mCNV size, decrease of network density, and shrinkage of CNV vessels with reservation of large diameter vessels 1 month after anti-VEGF treatment. Giorno et al. (29) described that the mCNV area and vessel area reduced by almost half after anti-VEGF therapy. Those results were consistent with the changes of qualitative features and quantitative biomarkers in OCTA images of our study.

It is noteworthy that vessel junction was the most dramatically changed biomarker after anti-VEGF therapy, decreasing by 50.36%. Meanwhile, junction density decreased by 32.31%. Choi et al. (17) considered junction points to indicate internal branching and anastomotic connections in neovascular networks in nAMD. Reinhard et al. (10) suggested that the vessel junction number and/or junction density could be a measure for angiogenic vessel sprouting in nAMD; active CNV lesions would be expected to present a higher vessel junction number and/or junction density. Takeuchi et al. (30) analyzed 15 consecutive treatment-naïve eyes with typical nAMD, and found junction density was also significantly reduced after anti-VEGF therapy, suggesting that immature vessels had reduced and that the maturation of vessels proceeded despite blocking the VEGF pathway. In our study, the sprouting activity and complexity of mCNV lesions represented by vessel junction and junction density were significantly lower in the post-IVI group. Vessel junction number was confirmed the most sensitive indicator in response to anti-VEGF agents and could possibly be used as an indicator of disease activity and a predictive factor to assess treatment responses.

Similarly, high value of total vessel length was another biomarker assumed to characterize active CNV lesions with abundant angiogenesis (31). Vessel length decreased significantly after IVI in our study, which also verified the decline of neovascular activity and effectiveness of anti-VEGF therapy. Furthermore, Al-Sheikh et al. (32) depicted that fractal dimension was higher in active nAMD CNV than in quiescent CNV and found a reduced fractal dimension after treatment. They proposed that the pattern of the CNV lesion after treatment might be less complex due to the attenuation of small-caliber vessels that may have a less significant effect on the vessel density compared with the large mature vessels. In our mCNV patients, fractal dimension also decreased after treatment, suggesting the weakening of vessel complexity.

It was interesting to notice that vessel diameter increased remarkably by 20.44% after IVI (*p* = 0.027). A possible explanation was that IVI pruned back the newly growing vessels but could not affect the pericyte-covered larger vessels, and the higher flow in the remaining vascular network would stimulate vessels to extend. This hypothesis was supported by many researchers, who suggested that anti-VEGF injection might trigger major feeder vessels to grow larger with fewer branching points and more vascular anastomotic connections by pruning of angiogenic vascular sprouts (30, 33, 34). In addition, vessel density revealed a slight increase by 1.82% after IVI with no significant difference, as vessel area and mCNV area reduced by 35.00 and 30.00%, respectively. Vessel tortuosity, as a biomarker of vessel complexity, was rarely mentioned in CNV research, but increased retinal venular tortuosity had been recognized as an important biomarker to indicate the progression of diabetic retinopathy stages (24). Vessel tortuosity also showed a slight increasement by 7.93% in post-IVI group in ours study with inadequate statistical significance (*p* = 0.276). Further studies with a larger population will help to determine the clinical significance of OCTA-based vessel density and vessel tortuosity in mCNV patients.

Anti-VEGF is a safe and efficacious treatment option for mCNV, requiring only a limited number of injections to obtain good anatomic and functional results (35). Our patients received an average of 2.19 ± 0.87 intravitreal injections. Similarly, patients with mCNV received an average of 2.9 intravitreal injections in MYRROR study (36). It is worth noting that the biomarker values of mCNV in “stable group (≤2 injections)” were lower than those in “unstable group (>2 injections)” except vessel density and junction density at baseline. Unfortunately, there were no statistical difference for all OCTA-based biomarkers between the “stable group” and “unstable group” (*p* > 0.05). Choi et al. (17) divided 71 nAMD eyes into the stable and unstable groups, and reported the OCTA-based biomarkers (CNV area, CNV density, vessel length, and junction density), except end points, were not different between the two groups. Roberts et al. (35) divided 25 eyes with nAMD into “good responder” group and “poor responders” group, and concluded that there was no significant difference between any of the microvascular quantitative features, whose results were similar to ours.

The lesion responses to anti-VEGF therapy were expressed by percent change of newly-established OCTA-based biomarkers and that of frequently-used OCT-based CRT, and the correlation analysis between the two categories was performed. There was a moderate correlation between CRT and mCNV area, vessel area, and vessel length (*r* = 0.552, *p* = 0.002; *r* = 0.518, *p* = 0.005; *r* = 0.440, *p* = 0.019). This was expected because of the initial definitions of these biomarkers closely associated with mCNV volumes. This result further validated the fundamental role of mCNV area and vessel area in OCTA quantitative analysis. A moderate correlation was also found between CRT and fractal dimension (*r* = 0.446, *p* = 0.017), indicating that the weakening of neovascular complexity partly accounted for the reduction of mCNV volume.

In our study, all of the biomarkers were measured in skeletonized binary OCTA images. Zhu et al. (22) used the same program to analyze the retinal vessels of diabetic retinopathy and reported that OCTA metrics obtained from skeletonized images were more effective than those from non-skeletonized images in detecting the retinal capillary. Segmentation errors were a challenging artifact for highly myopic patients, large chorioretinal atrophy areas, bad fixation, very long axial length, and deep posterior staphyloma induce inaccurate automatic layer segmentation on OCTA images. It is worth mentioning that manual segmentation in OCTA volumes increased sensitivity for CNV detection further from 53% to 92% (37). Arya et al. (21) also suggested that, automated segmentation algorithms of commercially available OCTA devices are limited in the identification and quantification of CNV lesions, and they emphasized the importance of manual adjustment of segmentation to visualize the full extent of CNV for detection and accurate area measurements. Consequently, we corrected correlation between the B-scan depth imaging and the OCTA by manually changing the boundaries of correctly contoured segmentation to obtain entire thickness mCNV.

The limitations of this study should be noted. Firstly, the follow-up period was relatively short and the number of subjects were relatively small. This may be one of the reasons why there was no statistical difference of the biomarkers between the “stable group” and “unstable group.” Furthermore, this is a retrospective study and there may be a selection bias, a prospective analysis with larger number of subjects would help validate our results. Finally, measurement accuracy might be compromised by the semiautomated approach used to obtain the OCTA-based biomarkers, and the manually selected CNV area.

In conclusion, OCTA, which simultaneously provides functional (optical coherence tomography angiograms) and morphological (OCT B-scans and *en face*) information, is a promising imaging modality that facilitates physicians to characterize mCNV lesion, assess, and predict therapeutic response, and plan the follow-up treatment. We applied semiautomated postprocessing approach to obtain binarized and skeletonized OCTA images of mCNV lesion for fully quantitative analysis to elucidate the morphological changes of mCNV after anti-VEGF therapy. Vessel junction was regarded as the most sensitive indicator of the mCNV activity, and assumed to be the most helpful biomarker to predict early therapeutic response to anti-VEGF therapy. Further, studies on development of fully automated quantitative methods and establishment of recognized OCTA-based biomarkers are required to improve the clinical applicability of OCTA quantitative analysis on mCNV.

## Data Availability Statement

The raw data supporting the conclusions of this article will be made available by the authors, without undue reservation.

## Ethics Statement

The study was approved by the ethics committee of the Second Affiliated Hospital, School of Medicine, Zhejiang University.

## Author Contributions

YW: conception, design, image interpretation, and manuscript preparation. XS: conception and design. ZH: data collection and analysis and manuscript preparation. TZ: development of the MATLAB program and image postprocessing. ZhiS: image interpretation and diagnosis. XF, JLin, ZC, ZhaS, PY, JM, LZ, JLi, LF, CS, and ZZ: patient treatment and data collection. All authors read and approved the final manuscript.

## Conflict of Interest

The authors declare that the research was conducted in the absence of any commercial or financial relationships that could be construed as a potential conflict of interest. The handling editor is currently organizing a Research Topic with one of the authors XS.
